# Simplified Histologic Mucosal Healing Scheme (SHMHS) for inflammatory bowel disease: a nationwide multicenter study of performance and applicability

**DOI:** 10.1007/s10151-022-02628-7

**Published:** 2022-06-01

**Authors:** A. Caputo, P. Parente, M. Cadei, M. Fassan, A. Rispo, G. Leoncini, G. Bassotti, R. Del Sordo, C. Metelli, M. Daperno, A. Armuzzi, V. Villanacci, The SHMHS Study Group

**Affiliations:** 1 Department of Advanced Biomedical Sciences, University of Naples, Naples, Italy; 2 Pathology Unit, Fondazione IRCCS Ospedale Casa Sollievo della Sofferenza, San Giovanni Rotondo, Foggia Italy; 3 Institute of Pathology, ASST Spedali Civili, Brescia, Italy; 4 Surgical Pathology Unit, Department of Medicine, University of Padua, Padua, Italy; 5 Department of Clinical Medicine and Surgery, Federico II University of Naples, Naples, Italy; 6 Pathology Unit, ASST del Garda, Desenzano del Garda, Brescia Italy; 7 Gastroenterology and Hepatology Section, Department of Medicine and Surgery, University of Perugia, Perugia, Italy; 8 Section of Anatomic Pathology and Histology, Department of Medicine and Surgery, Medical School, University of Perugia, Perugia, Italy; 9 Division of Gastroenterology, Ospedale Ordine Mauriziano di Torino, Turin, Italy; 10 IBD Unit, Presidio Columbus Fondazione Policlinico Universitario A. Gemelli IRCCS, Università Cattolica del Sacro Cuore, Rome, Italy

**Keywords:** Mucosal healing, Histological assessment, Endoscopic score, Inflammatory bowel disease

## Abstract

**Background:**

Assessment of mucosal healing is important for the management of patients with inflammatory bowel disease (IBD), but endoscopy can miss microscopic disease areas that may relapse. Histological assessment is informative, but no single scoring system is widely adopted. We previously proposed an eight-item histological scheme for the easy, fast reporting of disease activity in the intestine. The aim of the present study was to evaluate the performance of our Simplified Histologic Mucosal Healing Scheme (SHMHS).

**Methods:**

Between April and May 2021 pathologists and gastroenterologists in Italy were invited to contribute to this multicenter study by providing data on single endoscopic–histological examinations for their IBD patients undergoing treatment. Disease activity was expressed using SHMHS (maximum score, 8) and either Simple Endoscopic Score for Crohn’s Disease (categorized into grades 0–3) or Mayo Endoscopic Subscore (range 0–3).

**Results:**

Thirty hospitals provided data on 597 patients (291 Crohn’s disease; 306 ulcerative colitis). The mean SHMHS score was 2.96 (SD = 2.42) and 66.8% of cases had active disease (score ≥ 2). The mean endoscopic score was 1.23 (SD = 1.05), with 67.8% having active disease (score ≥ 1). Histologic and endoscopic scores correlated (Spearman’s ρ = 0.76), and scores for individual SHMHS items associated directly with endoscopic scores (chi-square *p* < 0.001, all comparisons). Between IBD types, scores for SHMHS items reflected differences in presentation, with cryptitis more common and erosions/ulcerations less common in Crohn’s disease, and the distal colon more affected in ulcerative colitis.

**Conclusions:**

SHMHS captures the main histological features of IBD. Routine adoption may simplify pathologist workload while ensuring accurate reporting for clinical decision making.

**Supplementary Information:**

The online version contains supplementary material available at 10.1007/s10151-022-02628-7.

## Introduction

Mucosal healing (MH) is a therapeutic target for inflammatory bowel disease (IBD), because it is associated with good clinical outcomes, including sustained clinical remission, low hospitalization rates, and reduced surgery and cancer rates [1, 2].⁠ Endoscopic MH, defined as the resolution of endoscopically visible mucosal inflammation and ulceration, does not necessarily reflect *microscopic* disease activity, which correlates better with relapse than do endoscopic findings alone [3].⁠ In fact, it has been reported that microscopic inflammation persists in as many as 25% of patients with endoscopically healed mucosa [3].⁠ In addition, in cases of IBD not responding to conventional therapy, a detailed histological mucosal assessment is needed to choose an appropriate alternative therapy, which in most cases consists in newer biological agents [4].⁠ An unequivocal, widely accepted definition of histological MH is, therefore, highly desirable.

The definition and scoring of histological MH are currently debated; at least 22 different histological scores for IBD have been devised [1]⁠ and until now used only in clinical trials [5–9].⁠ These scoring systems take into consideration even minimal deviations of mucosal, glandular, inflammatory and other lamina propria components, and thus have numerous histological variables, each of which is scored on an analog scale with many categories. These scales have multiple limitations, including a lack of full validation and heterogeneity in their thresholds for distinguishing quiescent disease and true histologic normalization [1, 6, 10].⁠ In addition, they have high interobserver variability, are difficult and time-consuming to use, and none has been validated histologically in clinical practice (Table 1) [1].⁠

**Table 1:** The main scores employed in histological assessment of inflammatory bowel disease

Simplified Geboes score		Nancy score		Robarts score	
Grade 0: No inflammatory activity	0.0 No abnormalities 0.1 Presence of architectural changes 0.2 Presence of architectural changes and chronic mononuclear infiltrate	Chronic inflammatory infiltrate (quantity of lymphocytes and plasma cells in the biopsy)	0 No increase 1 Mild but unequivocal increase 2 Moderate increase 3 Marked increase	Chronic inflammatory infiltrate	0 No increase 1 Mild but unequivocal increase 2 Moderate increase 3 Marked increase
Grade 1: Basal plasma cells	1.0 No increase 1.1.Mild increase 1.2 Marked increase	Neutrophils in the epithelium	0 None 1 < 50% crypt involved 2 > 50% crypt involved	Lamina propria neutrophils	0 None 1 Mild but unequivocal increase 2 Moderate increase 3 Marked increase
Grade 2A: Eosinophils in lamina propria	2A.0 No increase 2A.1 Mild increase 2A.2 Marked increase	Ulceration (visible epithelial injury, regeneration, fibrin, granulation tissue)	0 Absent 1 Present		
				Neutrophils in the epithelium	0 None 1 < 5% crypts involved 2 < 50% crypts involved 3 > 50% crypts involved
Grade 2B: Neutrophils in lamina propria	2B.0 No increase 2B.1 Mild increase 2B.2 Marked increase	Acute inflammatory cell infiltrate	0 None 1 Mild 2 Moderate 3 Severe		
				Erosion or ulceration	0 No erosion, ulceration or granulation tissue 1 Recovering epithelium + adjacent inflammation 1 Probable erosion-focally stripped 2 Unequivocal erosion 3 Ulcer or granulation tissue
Grade 3: Neutrophils in epithelium	3.0 None 3.1 < 50% crypts involved 3.2 > 50% crypts involved	Mucin depletion	0 None 1 Mild 2 Moderate 3 Severe		
Grade 4: Epithelial injury (in crypt and surface epithelium	4.0 None 4.1 Marked attenuation 4.2 Probable crypt destruction: probable erosions 4.3 Unequivocal crypt destruction: unequivocal erosions 4.4 Ulcer or granulation tissue	Neutrophils in lamina propria	0 None 1 Mild 2 Moderate 3 Severe		
		Basal plasmacytosis	0 None 1 Mild 2 Moderate 3 Severe		
		Serrated architecture (defined as the presence of dilated crypts showing a scalloped lumen)	0 None 1 < 5% crypt involved 2 < 50% crypt involved 3 > 50% crypt involved		

Some of the above-mentioned problems are due to the use of inappropriate morphological criteria for IBD in remission (quiescent or inactive disease) and inaccurate definitions of MH. For instance, while the presence of neutrophils in the lamina propria and crypts is a clear marker of active disease [1],⁠ the role of basal plasmacytosis in this regard is not as clear. Because basal plasmacytosis is known to have high predictive value for the first diagnosis of IBD [7, 8, 11, 12]⁠ and for the differential diagnosis with other forms of colitis [13],⁠ it has been hypothesized that an absence of basal plasmacytosis is required in healed IBD [14].⁠ This is contradictory, because the presence of basal plasma cells in this phase of disease is a sign of pre-existing IBD, and the number of plasma cells needed to document this feature is not known. Eosinophils, intermingled with plasma cells, are present at variable frequency in active and quiescent IBD [15, 16].⁠ Therefore, the presence of eosinophils does not rule out inactive disease.

In routine clinical practice, the evaluation of colonic biopsies should be carried out according to recommended best practice. As indicated by the European Crohn’s and Colitis Organisation (ECCO) and the European Society of Pathology (ESP), “for a reliable diagnosis of inflammatory bowel disease, ileocolonoscopy rather than rectoscopy should be performed. A minimum of two biopsies from at least five sites along the colon, including the rectum, and the terminal ileum should be obtained” (ECCO-ESP statement 1) [11].⁠ The evaluation should also take into consideration all the available clinical and endoscopic data. Adequate, correctly oriented biopsies are of paramount importance [17]. Histologically, the presence of neutrophils must be considered the distinctive sign of active disease, to differentiate from quiescent disease. This sign can also be used to express the efficacy of therapy (i.e., an absence of neutrophils indicates histological MH). Finally, for best interobserver agreement in the evaluation of the colonic mucosa, morphological scoring should be avoided, because it is complicated and subjective [18].⁠

A simplified histological MH scheme for assessing IBD patients undergoing treatment has been described [18].⁠ The scheme is designed to be used for patients with either Crohn’s disease (CD) or ulcerative colitis (UC). We showed, in a series of 24 patients, that the scheme gave similar results to other more commonly used scales, had high inter-rater agreement (k = 0.94), and was faster to calculate than other scales [18].⁠ It specifies the number of biopsies to examine, and offers the advantage of indicating the precise topographic localization of active and quiescent areas of disease. The present multicenter study assessed the performance and applicability of this scheme, the “Simplified Histological MH Scheme” (SHMHS). In particular, in a large sample of IBD cases, we tested the correlation between histologic and endoscopic scores. Moreover, we examined how individual items of the SHMHS vary between CD and UC patients and associate with disease-specific endoscopic scores.

## Materials and methods

This multicenter study reviewed endoscopic, histologic and clinical data of IBD patients. Pathologists and gastroenterologists belonging to the Italian Group for the Study of Inflammatory Bowel Disease (IG-IBD) and the Italian Group of Gastrointestinal Pathologists (GIPAD) were invited via email to contribute to the study between April and May 2021. The invitation was sent to 207 people, of which 30 responded and are acknowledged as study contributors at the end of this article.

Contributors were trained in the application of the scheme and were then requested to submit anonymized data from 20 IBD patients in treatment under their care. Inclusion criteria for the patients were: an established diagnosis of CD or UC; any ongoing or previous treatment for IBD; and proper sampling consisting in at least two endoscopic biopsies per segment (from cecum to rectum for UC; from ileum to rectum for CD). Data were collected via email using a structured form (Supplementary file 1). Each contributor was blinded to the data submitted by others. Contributors who submitted forms with missing data were contacted and requested to amend the form, which they did in all cases.

For each case, data were collected from clinical records regarding: age in years at the date of endoscopy, sex, diagnosis (CD or UC), and either the Mayo Clinic Endoscopic Subscore [19]⁠ for UC patients or the Simple Endoscopic Score for Crohn's Disease (SES-CD) [20]⁠ for CD patients. The Mayo Clinic Endoscopic Subscore was used unchanged (range 0–3), while the SES-CD (range 0–60) was summarized by the contributors on a scale of 0–3 using an online tool (https://www.igibdscores.it/it/score-sescd.html) as follows: a SES-CD score from 0 to 2 indicated disease in remission (grade 0); a score of 3–6 indicated mild endoscopic activity (grade 1); a score of 7–15, moderate endoscopic activity (grade 2); and a score > 15, severe endoscopic activity (grade 3).

**Fig. 1:**
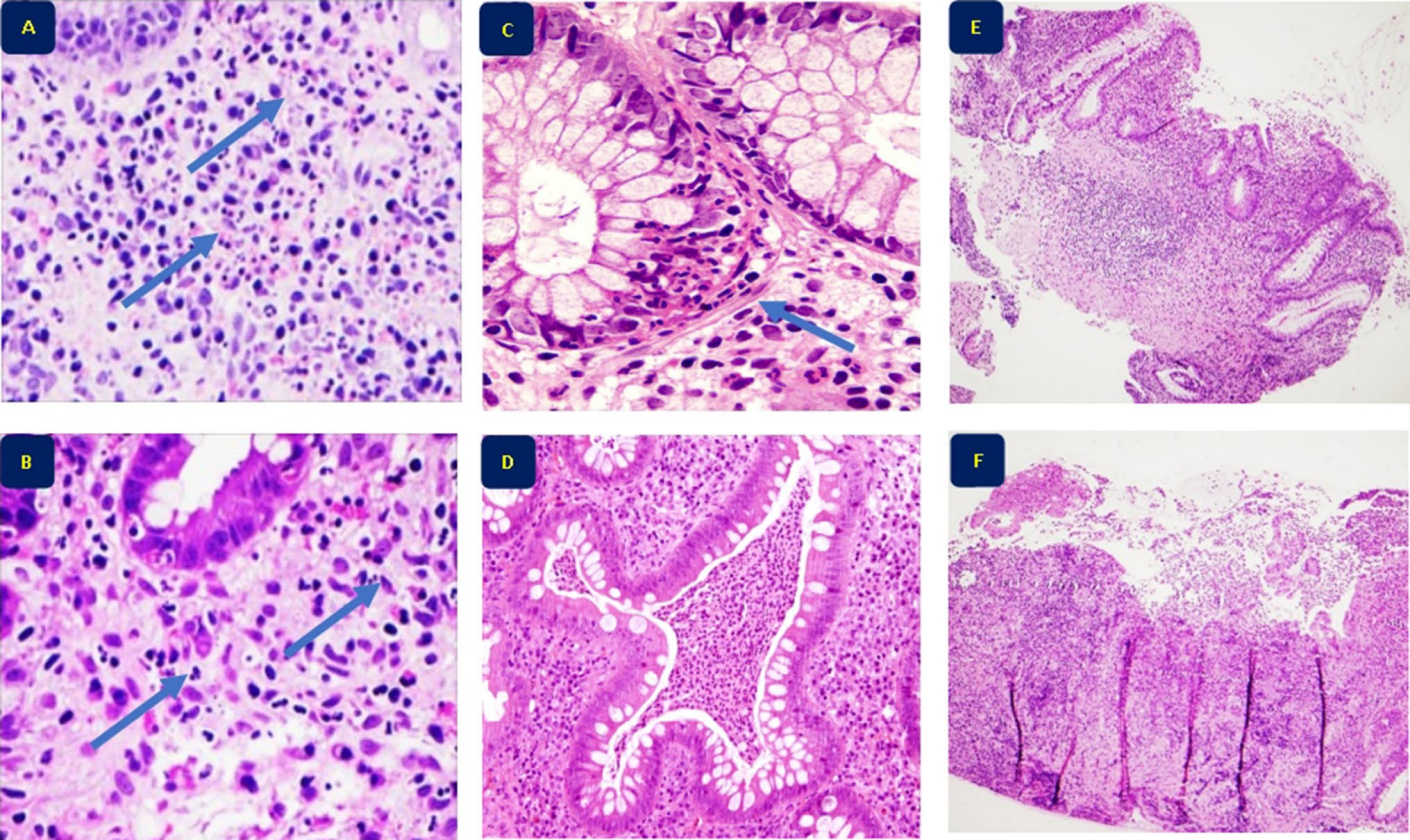
Histological features of inflammatory bowel disease investigated with Part I of the Simplified Histologic Mucosal Healing Scheme. **A**, **B**: Infiltration of neutrophils in lamina propria (arrows) (H&E × 400). **C** Cryptitis (arrow) (H&E × 400). **D** Crypt abscess (H&E × 400). **E**, **F** Erosions and ulcerations of the colonic mucosa (H&E × 40)

The SHMHS consists of two parts with a total of eight questions (Table 2 and Fig. 1). The questions are short, simple, and easy to score. Part I inquires about overall disease features with three questions, each scored as present (1 point) or absent (0 points); a score of “present” is indicated when at least one biopsy from any segment shows the feature. Part II addresses disease topography with one question per intestinal segment (right, transverse and descending colon; sigmoid colon and rectum; and, in CD patients only, ileum). These segments are scored as active (1 point), quiescent (0 points) or not involved (0 points). Active disease is selected when at least one biopsy from the segment identifies neutrophils in any location (lamina propria, crypts, or superficial epithelium). Quiescent disease is selected when at least one biopsy from the segment shows signs of chronic disease (e.g., crypt distortion, basal plasmacytosis) without signs of activity (i.e., neutrophils, erosions or ulcers). The total score ranges from 0 to 8, and a score ≥ 2 indicates histologically active disease.

**Table 2:** Simplified histological mucosal healing scheme (modified from Villanacci et al. [18]⁠)

Question	Value	Score
Part I. Features		
Neutrophils in the lamina propria	Present	1
	Absent	0
Cryptitis or crypt abscesses (presence of neutrophils)	Present	1
	Absent	0
Erosions or ulcerations (presence of granulation tissue)	Present	1
	Absent	0
Part II. Sites of involvement		
Ileum (CD patients only)	Active	1
	Quiescent	0
	Not involved	0
Right colon	Active	1
	Quiescent	0
	Not involved	0
Transverse colon	Active	1
	Quiescent	0
	Not involved	0
Descending colon	Active	1
	Quiescent	0
	Not involved	0
Sigmoid colon and rectum	Active	1
	Quiescent	0
	Not involved	0
Total score (range 0–8)

A central review of histologic scoring from four participating centers comprising 82 cases was performed to assess the interobserver concordance. All slides were re-examined by one author (VV) and the resulting score was compared to the one produced by the referring pathologist.

### Statistical analysis

A database was assembled and maintained using LibreOffice v7.2.0. Data were gathered from the contributors and inserted into the database centrally by one of the authors (AC) and analyzed using the R programming language v4.1.0 [21].⁠ Correlations between variables were assessed using Spearman’s test. Inferential statistics on categorical variables employed the Chi-squared test. The interobserver concordance was assessed using Cohen’s kappa with linear weights. A *p* value < 0.05 was taken as the threshold of statistical significance.

### Ethical considerations

Ethical approval for the study was granted by the institutional review board of Spedali Civili and University of Brescia (approval number NP4700, 22 April 2021). All patients (or the guardians of patients aged < 18 years) provided informed consent for the collection of their clinical data for medical purposes, and the institutional review board waived the need for further informed consent. The data were anonymized by each contributor before transmission.

## Results

A total of 597 cases (291 CD and 306 UC) were obtained from 30 hospitals throughout Italy, with a median of 20 cases per center. Overall, the patients had a mean age of 45.8 (± 17.0) years (Table 3), but the mean age for CD cases was slightly lower than that for UC cases (42.7 vs. 48.7 years). Slightly more than half of the population was male (54.6%). The average SHMHS score was 2.96 (SD = 2.42; range 0–8), with 399 cases (66.8%) having a score ≥ 2 and considered as having histologically active disease. The mean endoscopic score was 1.23 (SD = 1.05), and 405 cases (67.8%) had a score ≥ 1 and thus had endoscopically active disease. Of the 291 CD patients, 102 (35.1%) had a categorized SES-CD grade of 0, indicative of endoscopic remission. Moreover, of the 306 UC patients, 90 (29.4%) were in endoscopic remission with a Mayo score of 0.

**Table 3:** Clinicopathological characteristics of the study population

Characteristic	Total (*n* = 597)	Crohn's disease (*n* = 291)	Ulcerative colitis (*n* = 306)
Age, years			
Mean (SD)	45.8 (17.0)	42.7 (15.9)	48.7 (17.6)
Range	4.0–85.0	11.0–78.0	4.0–85.0
Sex, *n* (%)			
Male	326 (54.6)	160 (55.0)	166 (54.2)
Female	271 (45.4)	131 (45.0)	140 (45.8)
SHMHS score, mean (SD)	2.96 (2.42)	2.90 (2.46)	3.02 (2.38)
SHMHS score ≥ 2, *n* (%)	399 (66.8)	195 (67.0)	204 (66.7)
Endoscopic score, mean (SD)^a^	1.23 (1.05)	1.15 (1.04)	1.31 (1.05)
Endoscopic score, *n* (%)^a^			
0	192 (32.2)	102 (35.1)	90 (29.4)
1	155 (26.0)	78 (26.8)	77 (25.2)
2	169 (28.3)	75 (25.8)	94 (30.7)
3	81 (13.6)	36 (12.4)	45 (14.7)

*SHMHS* Simplified Histological Mucosal Healing Scheme

^a^Mayo Clinic Endoscopic Subscore for ulcerative colitis and SES-CD for Crohn’s disease (categorized into grades 0–3)

**Fig. 2:**
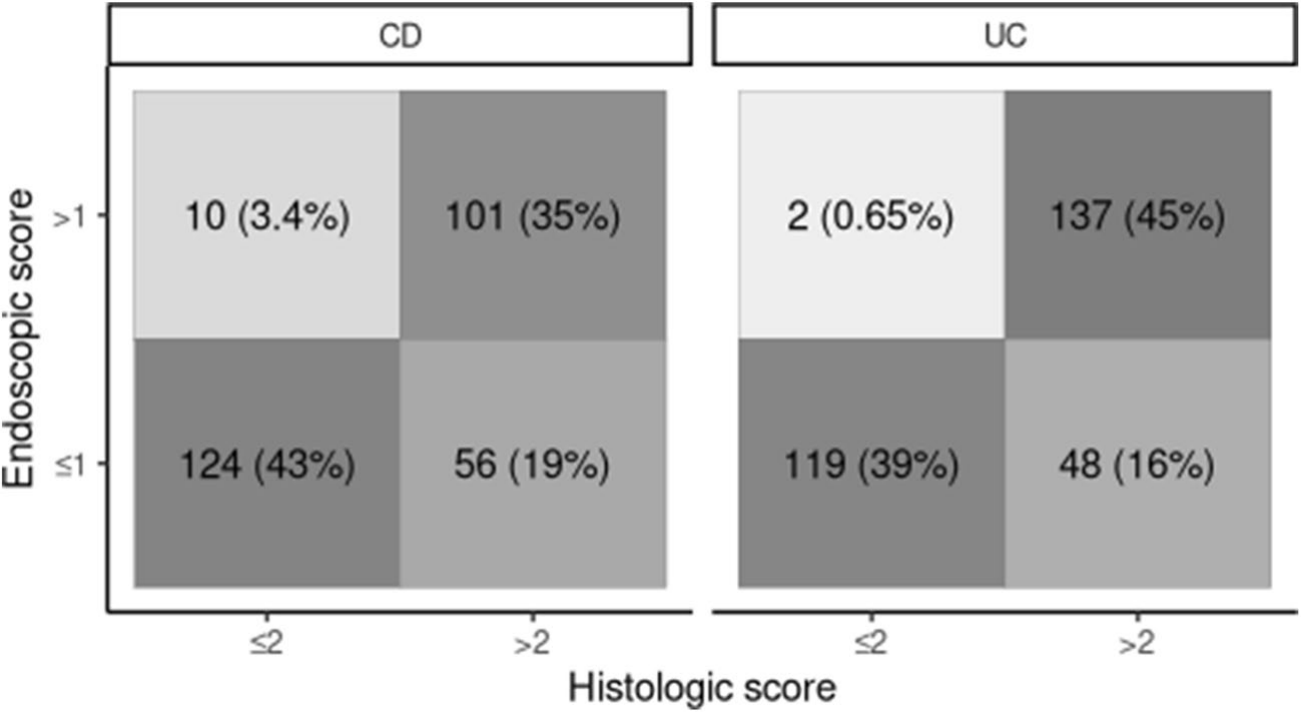
Confusion matrices showing the concordance between the SHMHS and the endoscopic scores (Mayo Clinic Endoscopic Subscore for ulcerative colitis patients; Simple Endoscopic Score for Crohn’s Disease). Values are shown as number (percentage) of patients*. SHMHS* Simplified Histologic Mucosal Healing Scheme, *CD* Crohn’s disease, *UC* ulcerative colitis

The histologic and endoscopic scores were strongly correlated in the study population as a whole (Spearman’s ρ = 0.76, *p* < 0.0001). Similar results were obtained for CD patients (ρ = 0.75) and UC patients (ρ = 0.76) separately (*p* < 0.0001 for both). These results indicate a strong albeit incomplete agreement between the two, as expected. In particular, concordant cases included 243 cases (40.7%) with an endoscopic score ≤ 1 and a SHMHS ≤ 2 and 238 cases (39.9%) with an endoscopic score > 1 and a SHMHS > 2; discordant cases included 12 cases (2.01%) with an endoscopic score > 1 but a SHMHS ≤ 2 and 104 cases (17.4%) with an endoscopic score ≤ 1 but a SHMHS > 2. Analysis of data from each center provided similar results, with a median ratio of concordant cases of 0.80 (interquartile range, 0.19). The confusion matrices for each of the two diseases (CD, UC) are shown in Fig. 2.

**Fig. 3:**
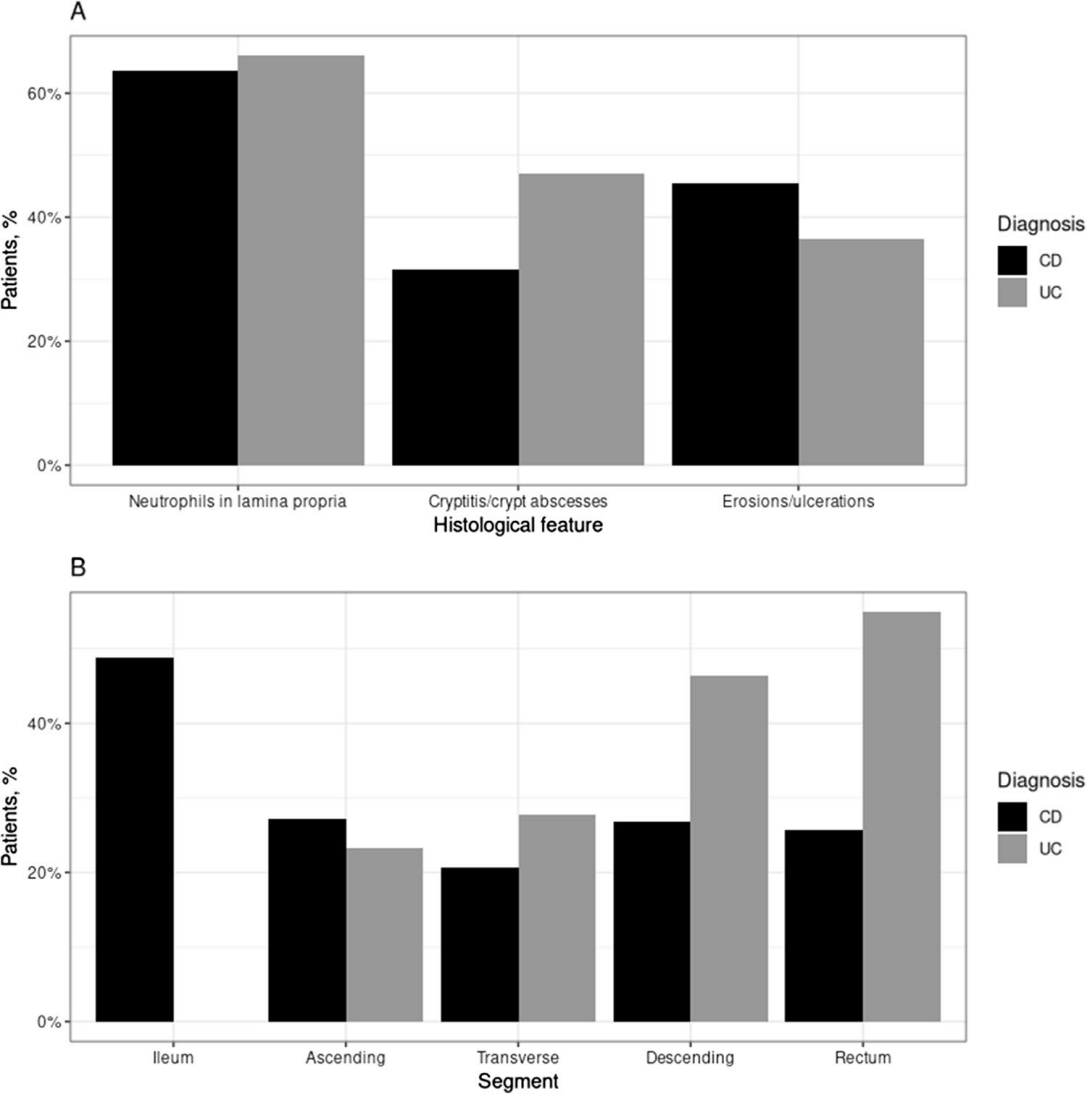
Individual SHMHS items compared between 291 CD patients and 306 UC patients**. A** Prevalence of the three histological features. **B** Prevalence of involvement of different intestinal segments. *CD* Crohn’s disease, *UC* ulcerative colitis

The two subgroups of patients were compared on single items on the SHMHS (Fig. 3). Regarding features of disease (Fig. 3A), cryptitis or crypt abscesses were less common in CD patients (31.6% vs. 47.1%, Chi-square *p* < 0.001), erosions or ulcerations were more common in CD patients (45.4% vs. 36.6%, *p* = 0.030), and neutrophils in the lamina propria were similarly prevalent in the two groups. Regarding the topography of involvement (Fig. 3B), similar percentages of CD and UC patients had involvement of the ascending colon, while for the other colonic segments (transverse, descending and sigmoid colon–rectum), UC patients were more frequently affected (*p* < 0.001 for all). These findings indicate a good capacity of the SHMHS to capture essential features of the two diseases.

Regarding the correspondence between individual items on the SHMHS and the endoscopic score. In CD patients (Table 4), we found a significant association between endoscopic score and each of the eight items of the histologic score (Pearson’s χ^2^, *p* < 0.001 for all). In UC patients (Table 5), we observed the same association (Pearson’s χ^2^, *p* < 0.001 for all). This suggests that the SHMHS is able to capture not only the essential histologic features of the two diseases, but also their different prevalence by endoscopic grade.

**Table 4:** Correspondence between individual items on the SHMHS and endoscopic score for 291 Crohn’s disease patients

SHMHS item	Endoscopic score, n (%)^a^				*p* value^b^
	0 (*n* = 102)	1 (*n* = 78)	2 (*n* = 75)	3 (*n* = 36)	
Neutrophils in lamina propria^c^	29 (28.4)	52 (66.7)	69 (92.0)	35 (97.2)	< 0.001
Cryptitis or crypt abscess^c^	10 (9.8)	18 (23.1)	43 (57.3)	21 (58.3)	< 0.001
Erosion or ulceration^c^	12 (11.8)	31 (39.7)	57 (76.0)	32 (88.9)	< 0.001
Ileum					
Not involved	18 (17.6)	6 (7.7)	7 (9.3)	2 (5.6)	< 0.001
Quiescent	71 (69.6)	26 (33.3)	14 (18.7)	5 (13.9)	
Active	13 (12.7)	46 (59.0)	54 (72.0)	29 (80.6)	
Ascending					
Not involved	37 (36.3)	37 (47.4)	19 (25.3)	4 (11.1)	< 0.001
Quiescent	55 (53.9)	28 (35.9)	27 (36.0)	5 (13.9)	
Active	10 (9.8)	13 (16.7)	29 (38.7)	27 (75.0)	
Transverse					
Not involved	50 (49.0)	40 (51.3)	27 (36.0)	5 (13.9)	< 0.001
Quiescent	47 (46.1)	30 (38.5)	27 (36.0)	5 (13.9)	
Active	5 (4.9)	8 (10.3)	21 (28.0)	26 (72.2)	
Descending					
Not involved	46 (45.1)	38 (48.7)	27 (36.0)	3 (8.3)	< 0.001
Quiescent	49 (48.0)	25 (32.1)	18 (24.0)	7 (19.4)	
Active	7 (6.9)	15 (19.2)	30 (40.0)	26 (72.2)	
Sigmoid-rectum					
Not involved	55 (53.9)	43 (55.1)	25 (33.3)	6 (16.7)	< 0.001
Quiescent	40 (39.2)	24 (30.8)	18 (24.0)	5 (13.9)	
Active	7 (6.9)	11 (14.1)	32 (42.7)	25 (69.4)	

*SHMHS* Simplified Histological Mucosal Healing Scheme

^a^Simple Endoscopic Score for Crohn’s Disease, categorized into grades 0–3; ^b^Chi-square test; ^c^Feature present

**Table 5:** Correspondence between individual items on the SHMHS and the endoscopic score for 306 ulcerative colitis patients

SHMHS item	Endoscopic score, *n* (%)^a^				*p* value^b^
	0 (*n* = 90)	1 (*n* = 77)	2 (*n* = 94)	3 (*n* = 45)	
Neutrophils in lamina propria^c^	20 (22.2)	45 (58.4)	93 (98.9)	44 (97.8)	< 0.001
Cryptitis or crypt abscess^c^	9 (10.0)	29 (37.7)	72 (76.6)	34 (75.6)	< 0.001
Erosion or ulceration^c^	5 (5.6)	17 (22.1)	59 (62.8)	31 (68.9)	< 0.001
Ascending					
Not involved	33 (36.7)	24 (31.2)	22 (23.4)	12 (26.7)	< 0.001
Quiescent	52 (57.8)	40 (51.9)	42 (44.7)	10 (22.2)	
Active	5 (5.6)	13 (16.9)	30 (31.9)	23 (51.1)	
Transverse					
Not involved	26 (28.9)	20 (26.0)	17 (18.1)	14 (31.1)	< 0.001
Quiescent	61 (67.8)	39 (50.6)	36 (38.3)	8 (17.8)	
Active	3 (3.3)	18 (23.4)	41 (43.6)	23 (51.1)	
Descending					
Not involved	9 (10.0)	6 (7.8)	4 (4.3)	2 (4.4)	< 0.001
Quiescent	76 (84.4)	43 (55.8)	21 (22.3)	3 (6.7)	
Active	5 (5.6)	28 (36.4)	69 (73.4)	40 (88.9)	
Sigmoid-rectum					
Not involved	6 (6.7)	3 (3.9)	1 (1.1)	0 (0)	< 0.001
Quiescent	77 (85.6)	37 (48.1)	10 (10.6)	4 (8.9)	
Active	7 (7.8)	37 (48.1)	83 (88.3)	41 (91.1)	

*SHMHS* Simplified Histological Mucosal Healing Scheme

^a^Mayo Clinic Endoscopic Subscore; ^b^Chi-square test; ^c^Feature present

Finally, the central revision of a subset of cases (*n* = 82) showed very minor discordances (one point in 17 cases, two points in 3 cases) with excellent interobserver concordance as assessed by Cohen’s kappa (κ = 0.894, *p* < 0.001).

## Discussion

This study evaluated the concordance between endoscopic MH and histological MH assessed with the SHMHS in patients with CD or UC. We found strong correlations between our histologic score and the disease-specific endoscopic scores. Regarding single items of our histologic scheme, results in CD and UC patient subgroups matched our understanding of IBD. For example, crypt abscesses were significantly more frequent in UC patients, erosions and ulcerations were more frequent in CD patients, and in terms of disease topography, the distal colon was more often involved in UC than CD patients. These findings suggest that, overall, the SHMHS is able to capture and summarize the essential histologic features of the two diseases and their different expression in the various disease stages.

The SHMHS emphasizes the role of neutrophils in defining activity or quiescence in IBD. Since minimal histological activity has been associated with the need for corticosteroid treatment and with relapse, including acute severe colitis requiring hospitalization [22],⁠ the SHMHS uses the most affected area (worst histological activity) of any single biopsy to define each case of “active” IBD.

Bryant et al. [1]⁠, on behalf of the International Organization of Inflammatory Bowel Disease, proposed that “the histological treatment target for UC or CD is to:

- induce absence of neutrophils (both in the crypts and lamina propria);
- induce the absence of basal plasma cells and ideally reduce lamina propria plasma cells to normal; c. reduce lamina propria eosinophils to normal.”

We agree with point (a). Regarding point (b), basal plasmacytosis is an excellent diagnostic marker at the moment of the first diagnosis and in cases of previously diagnosed IBD [13, 23].⁠ Some authors found an association between basal plasmacytosis and disease relapse [2, 13],⁠ but to date there is no exact definition of the amount of plasma cells and the number of biopsies to examine that indicates the risk of relapse. Furthermore, basal plasmacytosis is seen only in a minority of cases of relapse and thus is a relatively insensitive marker [15].⁠ Concerning point (c), there is only limited data on the association between eosinophils and relapse. Recently, Vande Casteele et al. [16]⁠ quantified eosinophils in colonic biopsies and found correlations between eosinophil density and both disease extent and corticosteroid therapy, but crucially not with histologic activity. These factors confirm our belief that neutrophils are the only real marker of activity in IBD.

The concept of “remission” in IBD patients has evolved over the past 20 years from simple clinical improvement to improvements on a set of clinical, laboratory and endoscopic tests. However, these three elements do not always agree with morphological findings [24, 25]. Failure to obtain biopsies from the terminal ileal mucosa in CD patients and from all colonic segments in UC patients can lead to an underestimation of residual IBD activity. We set our inclusion criteria strictly so that terminal ileal biopsies were mandatory in CD cases.

We observed a strong but not complete correlation between the histologic and endoscopic scores. This was expected, because one study reported cases that appeared quiescent at endoscopy but were active histologically [24].⁠ That study linked these discrepancies to a higher risk and frequency of relapse. This is an important clinical practice point: despite clinical and endoscopic remission, histology is of paramount importance and should always be examined to identify these patients and thus provide them with optimal care. A histologic score lower than expected from the endoscopic appearance might be explained by endoscopic overestimation of the grade or by sampling problems (e.g., sampled areas were quiescent but the disease was active elsewhere). Furthermore, the SHMHS takes only activity (neutrophils) into account, whereas endoscopic scores such as the SES-CD are based on chronic features as well.

This study has several limitations. First, it was observational and cross-sectional, so the evolution of histological features over time was not assessed. In addition, to accommodate the large sample size, we did not measure the time taken by each pathologist to diagnose each case. Furthermore, we were able to centrally review the histologic scoring only for a subset of cases (*n* = 82, 13.7%). Nevertheless we found excellent concordance. Conversely, this study has several strengths, including the strict inclusion criteria which ensured proper endoscopic sampling, the large population and the multicenter design.

## Conclusions

This study found that the SHMHS: (1) is able to capture the essential histologic features of IBD activity, (2) correlates well but not perfectly with the endoscopic picture, and (3) is able to highlight cases in which endoscopy is deceptively normal and residual disease activity can only be assessed histologically. We hope that, on the basis of this experience and differently from the other scores used only in trials, the SHMHS will be adopted worldwide in the routine histological diagnosis of IBD, to measure histological MH better, and ultimately improve care for IBD patients.

## Supplementary Information

Below is the link to the electronic supplementary material.

Supplementary file1 (XLSX 14 KB)

## Data Availability

The data underlying this article will be shared on reasonable request to the corresponding author.

## Appendix

Simplifed Histologic Mucosal Healing Scheme (SHMHS) study group participants, all based in Italy: Davide Giuseppe Ribaldone and Marta Vernero, Department of Medical Sciences, University of Torino, Turin; Federica Grillo and Luca Mastracci, Department of Pathology, University of Genoa, Genoa; Chiara Viganò, UOC Gastroenterologia, ASST Monza Ospedale San Gerardo, Monza; Giulia Scardino, Department of Gastroenterology, Ospedale Valduce, Como; Stefania Gambini, Department of Pathology, ASST Melegnano-Martesana, Milan; Francesca Boni, Department of Gastroenterology, ASST Melegnano-Martesana, Milan; Federica Furfaro, Department of Gastroenterology, IRCCS Humanitas, Milan; Cristina Bezzio and Simone Saibeni, Department of Gastroenterology, Ospedale di Rho, Milan; Michela Campora, Department of Pathology, Ospedale Santa Chiara, Trento; Eliana Greco, Edoardo V. Savarino and Fabiana Zingone, Department of Surgery, Oncology and Gastroenterology – DiSCOG, University of Padua, Padua; Daniele Canova, UOC di Gastroenterologia, AULSS 8 Berica, Vicenza; Irene Coati, Department of Pathology, Ospedale dell'Angelo, Mestre (VE); Davide Checchin, Department of Gastroenterology, Ospedale dell'Angelo, Mestre (VE); Marta Gobbato, Department of Pathology, Ospedale di Feltre, Belluno; Antonio Ferronato, UOSVD Endoscopia Digestiva, PO Alto Vicentino, Santorso (VI); Alice Morini, Department of Pathology, PO Alto Vicentino, Santorso (VI); Michele Campigotto, Department of Medicine and Surgery, University of Trieste, Trieste; Maria Raffaella Ambrosio, UOC Anatomia Patologica, Azienda Toscana Nord Ovest, Massa; Andrea Sbrozzi-Vanni, UOC Endoscopia Digestiva, Azienda Toscana Nord Ovest, Massa; Francesca De Nigris, Nicola Libertà Decarli, Martina Giannotta and Andrea Nucci, Department of Gastroenterology, USL Centro Toscana, Florence; Stefano Lazzi, Department of Medical Biotechnology, University of Siena, Siena; Marco Valvano, Department of Gastroenterology, P.O. S. Salvatore, L'Aquila; Ambra Magiotta, Department of Gastroenterology, AOU Sant’Andrea, Rome; Chiara Taffon, Department of Pathology, Campus Biomedico, Rome; Paola Balestrieri, Department of Gastroenterology, Campus Biomedico, Rome; Fabrizio Bossa, Department of Gastroenterology, IRCCS Ospedale Casa Sollievo della Sofferenza, Foggia; Gerardo Cazzato, Anna Colagrande, Antonio d’Amati, Giuseppe Ingravallo, Domenico Piscitelli and Luciana Scuccimarri, Department of Pathology, Policlinico di Bari, Bari; Rocco Spagnuolo, Department of Gastroenterology, University Magna Graecia, Catanzaro; Barbara Scrivo, UOC Gastroenterologia ed Endoscopia digestiva, Arnas Civico Di Cristina Benfratelli, Palermo; Walter Fries and Anna Viola, Department of Gastroenterology, AOU Policlinico di Messina, Messina.

## References

[1] Bryant RV, Winer S, Travis SP, Riddell RH (2014). Systematic review: histological remission in inflammatory bowel disease. Is ‘complete’ remission the new treatment paradigm? An IOIBD initiative. J Crohn’s Colitis.

[2] Bitton A, Peppercorn MA, Antonioli DA (2001). Clinical, biological, and histologic parameters as predictors of relapse in ulcerative colitis. Gastroenterology.

[3] Pennelli G, Grillo F, Galuppini F (2020). Gastritis: update on etiological features and histological practical approach. Pathologica.

[4] Mosli MH, Feagan BG, Zou G (2017). Development and validation of a histological index for UC. Gut.

[5] Mazzuoli S, Guglielmi FW, Antonelli E (2013). Definition and evaluation of mucosal healing in clinical practice. Dig Liver Dis.

[6] Mosli MH, Feagan BG, Zou G (2015). Reproducibility of histological assessments of disease activity in UC. Gut.

[7] Langner C, Magro F, Driessen A (2014). The histopathological approach to inflammatory bowel disease: a practice guide. Virchows Arch.

[8] Lang-Schwarz C, Agaimy A, Atreya R (2021). Maximizing the diagnostic information from biopsies in chronic inflammatory bowel diseases: recommendations from the Erlangen International Consensus Conference on Inflammatory Bowel Diseases and presentation of the IBD-DCA score as a proposal for a new i.. Virchows Arch.

[9] Marchal-Bressenot A, Salleron J, Boulagnon-Rombi C (2017). Development and validation of the Nancy histological index for UC. Gut.

[10] Villanacci V, Antonelli E, Geboes K (2013). Histological healing in inflammatory bowel disease: a still unfulfilled promise. World J Gastroenterol.

[11] Magro F, Langner C, Driessen A (2013). European consensus on the histopathology of inflammatory bowel disease. J Crohn’s Colitis.

[12] Stange EF, Travis SPL, Vermeire S (2008). European evidence-based consensus on the diagnosis and management of ulcerative colitis: definitions and diagnosis. J Crohn’s Colitis.

[13] Bessissow T, Lemmens B, Ferrante M (2012). Prognostic value of serologic and histologic markers on clinical relapse in ulcerative colitis patients with mucosal healing. Am J Gastroenterol.

[14] Pai RK, Geboes K (2018). Disease activity and mucosal healing in inflammatory bowel disease: a new role for histopathology?. Virchows Arch.

[15] Farkas K, Reisz Z, Sejben A (2016). Histological activity and basal plasmacytosis are nonpredictive markers for subsequent relapse in ulcerative colitis patients with mucosal healing. J Gastroenterol Pancreatol Liver Disord.

[16] Vande Casteele N, Leighton JA, Pasha SF (2021). Utilizing deep learning to analyze whole slide images of colonic biopsies for associations between eosinophil density and clinicopathologic features in active ulcerative colitis. Inflamm Bowel Dis.

[17] Reggiani Bonetti L, Leoncini G, Daperno M (2021). Histopathology of non-IBD colitis practical recommendations from pathologists of IG-IBD Group. Dig Liver Dis.

[18] Villanacci V, Antonelli E, Lanzarotto F (2017). Usefulness of different pathological scores to assess healing of the mucosa in inflammatory bowel diseases: a real life study. Sci Rep.

[19] Schroeder KW, Tremaine WJ, Ilstrup DM (1987). Coated oral 5-aminosalicylic acid therapy for mildly to moderately active ulcerative colitis. N Engl J Med.

[20] Daperno M, D’Haens G, Van Assche G (2004). Development and validation of a new, simplified endoscopic activity score for Crohn’s disease: the SES-CD. Gastrointest Endosc.

[21] R Core Team R: A language and environment for statistical computing

[22] Bryant RV, Burger DC, Delo J (2016). Beyond endoscopic mucosal healing in UC: histological remission better predicts corticosteroid use and hospitalisation over 6 years of follow-up. Gut.

[23] Villanacci V, Antonelli E, Reboldi G (2014). Endoscopic biopsy samples of naïve “colitides” patients: role of basal plasmacytosis. J Crohns Colitis.

[24] Rosenberg L, Nanda KS, Zenlea T (2013). Histologic markers of inflammation in patients with ulcerative colitis in clinical remission. Clin Gastroenterol Hepatol.

[25] Fiorino G, Cesarini M, Indriolo A, Malesci A (2011). Mucosal healing in ulcerative colitis: where do we stand?. Curr Drug Targets.

