# Correction to: Simplified Histologic Mucosal Healing Scheme (SHMHS) for inflammatory bowel disease: a nationwide multicenter study of performance and applicability

**DOI:** 10.1007/s10151-022-02714-w

**Published:** 2022-10-26

**Authors:** A. Caputo, P. Parente, M. Cadei, M. Fassan, A. Rispo, G. Leoncini, G. Bassotti, R. Del Sordo, C. Metelli, M. Daperno, A. Armuzzi, V. Villanacci, The SHMHS Study Group

**Affiliations:** 1 Department of Advanced Biomedical Sciences, University of Naples, Naples, Italy; 2 Pathology Unit, Fondazione IRCCS Ospedale Casa Sollievo della Sofferenza, San Giovanni Rotondo, Foggia Italy; 3 Institute of Pathology, ASST Spedali Civili, Brescia, Italy; 4 Surgical Pathology Unit, Department of Medicine, University of Padua, Padua, Italy; 5 Department of Clinical Medicine and Surgery, Federico II University of Naples, Naples, Italy; 6 Pathology Unit, ASST del Garda, Desenzano del Garda, Brescia Italy; 7 Gastroenterology and Hepatology Section, Department of Medicine and Surgery, University of Perugia, Perugia, Italy; 8 Section of Anatomic Pathology and Histology, Department of Medicine and Surgery, Medical School, University of Perugia, Perugia, Italy; 9 Division of Gastroenterology, Ospedale Ordine Mauriziano di Torino, Turin, Italy; 10 IBD Unit, Presidio Columbus Fondazione Policlinico Universitario A. Gemelli IRCCS, Università Cattolica del Sacro Cuore, Rome, Italy

## Correction to: Techniques in Coloproctology (2022) 26:713–723 10.1007/s10151-022-02628-7

In the original publication, the study group author names are listed under Appendix section and it should be listed in Acknowledgement section as follows.

**Acknowledgements**

Simplified Histologic Mucosal Healing Scheme (SHMHS) study group participants, all based in Italy: Davide Giuseppe Ribaldone and Marta Vernero, Department of Medical Sciences, University of Torino, Turin; Federica Grillo and Luca Mastracci, Department of Pathology, University of Genoa, Genoa; Chiara Viganò, UOC Gastroenterologia, ASST Monza Ospedale San Gerardo, Monza; Giulia Scardino, Department of Gastroenterology, Ospedale Valduce, Como; Stefania Gambini, Department of Pathology, ASST Melegnano-Martesana, Milan; Francesca Boni, Department of Gastroenterology, ASST Melegnano-Martesana, Milan; Federica Furfaro, Department of Gastroenterology, IRCCS Humanitas, Milan; Cristina Bezzio and Simone Saibeni, Department of Gastroenterology, Ospedale di Rho, Milan; Michela Campora, Department of Pathology, Ospedale Santa Chiara, Trento; Eliana Greco, Edoardo V. Savarino and Fabiana Zingone, Department of Surgery, Oncology and Gastroenterology – DiSCOG, University of Padua, Padua; Daniele Canova, UOC di Gastroenterologia, AULSS 8 Berica, Vicenza; Irene Coati, Department of Pathology, Ospedale dell'Angelo, Mestre (VE); Davide Checchin, Department of Gastroenterology, Ospedale dell'Angelo, Mestre (VE); Marta Gobbato, Department of Pathology, Ospedale di Feltre, Belluno; Antonio Ferronato, UOSVD Endoscopia Digestiva, PO Alto Vicentino, Santorso (VI); Alice Morini, Department of Pathology, PO Alto Vicentino, Santorso (VI); Michele Campigotto, Department of Medicine and Surgery, University of Trieste, Trieste; Maria Raffaella Ambrosio, UOC Anatomia Patologica, Azienda Toscana Nord Ovest, Massa; Andrea Sbrozzi-Vanni, UOC Endoscopia Digestiva, Azienda Toscana Nord Ovest, Massa; Francesca De Nigris, Nicola Libertà Decarli, Martina Giannotta and Andrea Nucci, Department of Gastroenterology, USL Centro Toscana, Florence; Stefano Lazzi, Department of Medical Biotechnology, University of Siena, Siena; Marco Valvano, Department of Gastroenterology, P.O. S. Salvatore, L'Aquila; Ambra Magiotta, Department of Gastroenterology, AOU Sant’Andrea, Rome; Chiara Taffon, Department of Pathology, Campus Biomedico, Rome; Paola Balestrieri, Department of Gastroenterology, Campus Biomedico, Rome; Fabrizio Bossa, Department of Gastroenterology, IRCCS Ospedale Casa Sollievo della Sofferenza, Foggia; Gerardo Cazzato, Anna Colagrande, Antonio d’Amati, Giuseppe Ingravallo, Domenico Piscitelli and Luciana Scuccimarri, Department of Pathology, Policlinico di Bari, Bari; Rocco Spagnuolo, Department of Gastroenterology, University Magna Graecia, Catanzaro; Barbara Scrivo, UOC Gastroenterologia ed Endoscopia digestiva, Arnas Civico Di Cristina Benfratelli, Palermo; Walter Fries and Anna Viola, Department of Gastroenterology, AOU Policlinico di Messina, Messina.

